# A field‐validated ensemble species distribution model of *Eriogonum pelinophilum*, an endangered subshrub in Colorado, USA

**DOI:** 10.1002/ece3.10816

**Published:** 2023-12-14

**Authors:** Scott N. Zimmer, Kenneth W. Holsinger, Carol A. Dawson

**Affiliations:** ^1^ Uncompahgre Field Office Bureau of Land Management Montrose Colorado USA; ^2^ Fire Sciences Laboratory Rocky Mountain Research Station, U.S. Forest Service Missoula Montana USA; ^3^ Colorado State Office Bureau of Land Management Lakewood Colorado USA

**Keywords:** clay‐loving wild buckwheat, endangered species, ensemble model, field validation, habitat suitability, LiDAR, model validation, species distribution model

## Abstract

Understanding the suitable habitat of endangered species is crucial for agencies such as the Bureau of Land Management to plan management and conservation. However, few species distribution models are directly validated, potentially limiting their application in management. In preparation for a Species Status Assessment of clay‐loving wild buckwheat (*Eriogonum pelinophilum*), an endangered subshrub found in southwest Colorado, we ran a series of species distribution models to estimate the species' potential occupied habitat and validated these models in the field. A 1‐meter resolution digital elevation model derived from LiDAR and a high‐resolution geology mapping helped identify biologically relevant characteristics of the species' habitat. We employed a weighted ensemble model based on two Random Forest and one Boosted Regression Tree model, and discrimination performance of the ensemble model was high (AUC‐PR = 0.793). We then conducted a systematic field survey of model habitat suitability predictions, during which we discovered 55 new subpopulations of the species and demonstrated that new species observations were strongly associated with model predictions (*p* < .0001, Cliff's delta = 0.575). We further refined our original models by incorporating the additional species occurrences collected in the field survey, a new explanatory variable, and a more diverse set of models. These iterative changes marginally improved performance of the ensemble model (AUC‐PR = 0.825). Direct validation of species distribution models is extremely rare, and our field survey provides strong validation of our model results. This helps increase confidence to utilize predictions in planning. The final model predictions greatly improve the Bureau of Land Management's understanding of the species' habitat and increase our ability to consider potential habitat in planning land use activities such as road development and travel management.

## Graphical abstract

We ran a series of species distribution models to determine the suitable habitat of an endangered species, *Eriogonum pelinophilum*. These models were then validated through field surveys, during which 55 new subpopulations were discovered and we demonstrated that new species observations were strongly associated with model predictions. This rigorous validation helps justify the model's use in land management planning.

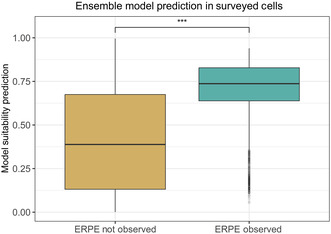

## 1 INTRODUCTION

Species distribution models (SDMs) are widely used tools to understand the primary habitat characteristics associated with species occurrence patterns (Guisan & Thuiller, 2005). These models can aid prioritization of conservation decisions (Guisan et al., 2013) and can guide further survey and management priorities (Graham et al., 2004; Hernandez et al., 2006). SDMs broadly compare habitat characteristics where a species has been observed to characteristics throughout a species' range to determine the conditions associated with species presence and predict potential habitat (Araújo & Guisan, 2006; Phillips et al., 2009).

SDMs have particular importance for rare, at‐risk, or endangered species. Estimation of a rare species' distribution is central to their assessment under International Union for Conservation of Nature guidelines (IUCN, 2001), and accurate predictions of the habitat of rare species is crucial for making informed conservation and management decisions (Ramirez‐Reyes, Nazeri, et al., 2021). At the same time, rare species pose unique challenges and opportunities for modeling. SDMs may have high accuracy if rare species occupy highly specialized habitats and limited geographic areas, but accuracy may also be limited by small population size and presence data gathered over long periods (Lomba et al., 2010; Sousa‐Silva et al., 2014).

There has been growing interest in using SDMs to guide field surveys, with some relevant examples found in a review (Fois et al., 2018), but given the ubiquity of SDMs these examples are still very uncommon. Furthermore, direct validation of model predictions is difficult and extremely rare (Araújo & Guisan, 2006), though some relevant examples exist (Halvorsen, 2012; Johnson et al., 2023; Searcy & Shaffer, 2014; Westwood et al., 2020; Williams et al., 2009). Model validation is a central tool to assess the predictive capabilities of models (Tredennick et al., 2021), and without robust model validation of SDMs their relevance and application for planning is unclear (Loiselle et al., 2003).

We ran a series of species distribution models and a weighted ensemble model of clay‐loving wild buckwheat (*Eriogonum pelinophilum*), an endangered species in western Colorado, United States. We then conducted a systematic field survey of the model predictions as a direct model validation procedure. This rare opportunity helped us discover 55 new subpopulations of the species and allowed us to directly validate our model, greatly increasing our confidence in using the model predictions in planning.

Our goal through this analysis was to obtain high‐resolution predictions of the species' potential habitat and better understand the ecology of the species to promote conservation. This was completed in preparation for a Species Status Assessment (SSA) of *E. pelinophilum*. The SSA process was developed by the U.S. Fish and Wildlife Service to inform all Endangered Species Act decisions, and is a standardized, repeatable analytical approach that provides a comprehensive analysis of the ecology, current condition, and expected future condition of at‐risk species (Smith et al., 2018). *E. pelinophilum* is a long‐lived perennial subshrub, generally growing 5–10 cm tall and 8–20 cm across (USFWS, 1988). The U.S. Fish and Wildlife Service determined that *E. pelinophilum* met the criteria of an endangered species and listed it under the Endangered Species Act in 1984, based on the species' small known population size, limited distribution, and land‐use conflicts in its range (USFWS, 1984).

## 2 METHODS

### 2.1 Study area

**FIGURE 1:**
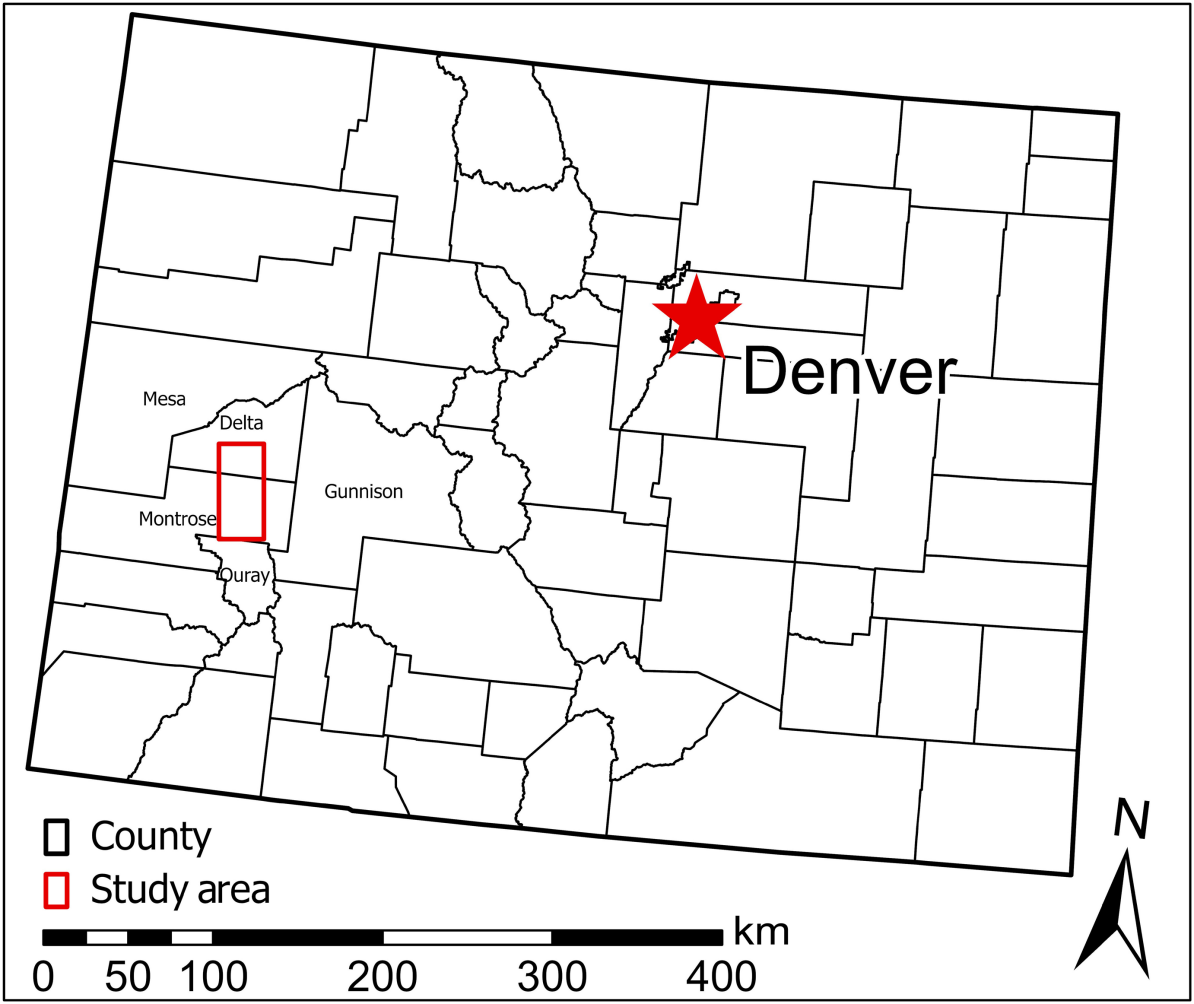
Map of the entire study area within western Colorado, within Delta and Montrose counties, with neighboring counties also labeled.

The study area was western Colorado, USA, including the entire known range of *E. pelinophilum* (hereafter, “ERPE”) (Figure 1). The species' known distribution encompasses a narrow band of badlands derived from the Mancos Shale formation east of U.S. Highway 50 between the towns of Delta and Montrose, in Delta and Montrose counties (Peterson, 1982). This formation is found on both private lands and Bureau of Land Management (BLM) lands managed by the Uncompahgre Field Office. Mancos Shale‐derived soils tend to have high clay and silt content, as well as high salt and selenium concentrations. These easily erodible soils form a variety of topographic features including steep badlands, gently rolling hills, alluvial fans, and flats.

Total annual precipitation in the study area ranges from an average of 22 cm at the northern extent of the range, to 32 cm at the southern extent (PRISM Climate Group, 2016). In the northern, lower elevation areas (1600 m above sea level), ERPE is thought to be more restricted to northerly aspects and swales that accumulate snow in the winter (O'Kane Jr, 1985). In the higher elevation (1900 m above sea level) southern extent of the range, the species is less restricted, but abundance tends to be greatest on northerly aspects (BLM, 2022a).

Plant communities associated with clay‐loving wild buckwheat are best described as an Inter‐Mountain Basins Mixed Salt Desert Scrub ecological system (Crawford et al., 2016). The vegetation community is characterized by an open shrubland and is predominantly comprised of halophytic shrubs and subshrubs including shadscale (*Atriplex confertifolia*), Gardner's saltbush (*Atriplex gardneri*), mat saltbush (*Atriplex corrugata*), budsage (*Artemisia spinescens*), and *Halogeton glomeratus*. Higher elevation sites display more heterogeneous black sagebrush (*Artemisia nova*) communities with *Achnatherum hymenoides*, *Xylorhiza venusta*, and another local endemic, Adobe Hills beardtongue (*Penstemon retrorsus*) (Neely, 1985; O'Kane, 1985).

### 2.2 Original model

#### 2.2.1 Input points

The original model included 1009 points of ERPE presence. The majority of these points (858) represented locations where point observations of individuals had been collected in the field by the BLM and others since the listing of the species in 1984. The remaining 151 points were manually generated within polygon observations also collected in the field which were geographically distinct from the point observations (typically separated by 1–2 km). The polygon observations represent larger, more continuous ERPE subpopulations that observers chose to map as polygons. Points were manually added in these polygons to obtain a more representative sample of conditions across the species' habitat in areas where no point observations were available. Multiple points were generated within larger polygons, but were not closer together than 10 m. The total 1009 ERPE presence points spanned much of the species' known range.

Background points were obtained by generating random points within the study area extent (Hefley & Hooten, 2016). A total of 43,334 random background points overlapping the predictor data were generated. Though some background points may be generated very close to ERPE presence points, the very large number of random points generated ensured that these points as a whole substantially differed from the presence points.

All analysis described in this paper was carried out in R 4.1.2 (R Core Team, 2021), with the ‘terra’ package (Hijmans et al., 2022) used for raster manipulation and ‘ggplot2’ package (Wickham & Chang, 2016) for plotting. Additional packages used in modeling are detailed below.

#### 2.2.2 Environmental covariates

Many of the covariates used in modeling were calculated from a 1‐m resolution LiDAR‐derived digital elevation model (DEM). This dataset was selected because of its extremely high resolution and ability to most accurately capture microtopography. In addition to elevation, we calculated slope, northness, and eastness from the DEM. Northness was calculated by generating aspect in degrees, then transforming as follows: *northness* = *cos* (*aspect* * *π*/180). Eastness was similarly calculated: *eastness* = *sin* (*aspect* * *π*/180). Transforming aspect into eastness and northness is necessary because aspect is circular (Clark et al., 1999).

We also included geologic formation as a covariate. Since we believed EPRE was associated with a single geologic formation (the Smoky Hill member of the Mancos Shale, formation "kms"), we generated a buffer of distance from this formation using 1:24 k layers of geology available in the majority of our study area (Morgan et al., 2007, 2008, Noe et al., 2007, 2013). We transformed this buffer distance into four classes rather than treating it as a continuous variable. One class included distances from 0 m (within formation "kms") up to 1 m away, the next included distances from 1 to 10 m away, then 10–100 m, and lastly farther than 100 m. One quadrangle of 1:24 k geology in the species' range, the Red Rock Canyon quadrangle, was not available. This area was omitted from analysis.

Lastly, we included 30‐year normal precipitation (1991–2020) from PRISM (PRISM Climate Group, 2016). Originally available at 800‐m resolution, we resampled precipitation to 1‐m resolution by bilinear interpolation to align cells of all predictors. Though this method creates interpolated data and does not truly increase data resolution, we employed it to preserve the high‐resolution LiDAR‐derived data and obtain more realistic precipitation values for prediction without sharp cell transitions (Phillips et al., 2006; Wang et al., 2006). Model variable coefficients derived from interpolated data may be suspect, but we do not report coefficients for this reason and because we mainly employed tree‐based methods without such coefficients.

We extracted the values of each of these environmental covariates onto the presence and background points, then used these values in modeling.

#### 2.2.3 Modeling

We randomly selected a subset of two‐thirds of both the presence and background points to include in modeling. This allowed one‐third of the points to serve as true model validation points which were never included in model construction. All modeling and validation was performed with these split training and validation data.

We ran four modeling approaches in our original modeling effort: two different implementations of Random Forest—a downsampled random forest from the ‘randomForest’ package (Liaw & Wiener, 2002) and a shallow random forest from the ‘ranger’ package (Wright et al., 2019)—and Boosted Regression Tree (BRT) and MaxEnt both fit in the ‘dismo’ package (Hijmans et al., 2017). These modeling methods are widely used in species distribution modeling (Valavi et al., 2022). In the downsampled random forest, each tree included a random subset of an equal number of presence and background points, while in the shallow random forest, each tree was restricted to a maximum depth of only two splits (Valavi et al., 2021).

From the models, we generated raster layers of habitat suitability predictions corresponding to all cells in the environmental covariate rasters. These represent predictions of species habitat suitability, which should be interpreted as similarity to the species' currently occupied habitat because current presence was the basis of our model (Latif et al., 2015).

We calculated many metrics of model accuracy. Many have identified AUC‐PR (area under the precision‐recall curve) as the most relevant for rare species (Sofaer et al., 2019). AUC‐ROC (area under the receiver operating characteristic curve) is typically presented as well, but this metric is more strongly influenced by true absences, which can overwhelm presences in models of rare species (Sofaer et al., 2019). Pearson's correlation between predicted suitability and occurrence observations has the advantage of addressing model value calibration (Phillips & Elith, 2010). Some have promoted similarity indices such as Sørensen's similarity index for model validation (Leroy et al., 2018). However, similarity indices require thresholding model results, which causes information loss and may lead to misleading results (Guillera‐Arroita et al., 2015; Lawson et al., 2014).

We used only threshold‐independent metrics for model validation (AUC‐PR, AUC‐ROC, and correlation) and used AUC‐PR to calculate a weighted ensemble model. The weighted ensemble was produced by first dividing each model's AUC‐PR by the sum of all models' AUC‐PR. This determined each model's weight in the ensemble. Each model was then multiplied by its respective weight, and then added to obtain the ensemble prediction (Ramirez‐Reyes, Nazeri, et al., 2021). We did not include MaxEnt in the ensemble model because its AUC‐PR was somewhat lower than the other models (Table 1).

**TABLE 1:** Validation metrics of models in the original model.

	AUC‐ROC	AUC‐PR	Correlation	Normalized average
RF Downsample	**0.989**	0.792	0.606	**0.924**
RF Shallow	0.987	0.773	0.547	0.537
BRT	**0.989**	0.728	**0.623**	0.874
MaxEnt	0.980	0.618	0.608	0.268
Weighted Ensemble	**0.989**	**0.793**	0.599	0.897

*Note*: The bolded number in each column denotes the model(s) with the highest score. Normalized average was calculated by scaling the data range of each validation metric from zero to one, then averaging the scaled result of the three metrics for each model.

To account for differences in performance across validation metrics, we calculated a normalized average of each model's validation scores. We did this by scaling the data range for each validation metric from 0 to 1, then averaging the scaled results across the metrics we utilized (AUC‐PR, AUC‐ROC, and correlation). This provided a single score for each model which considered performance in all three validation metrics.

We applied a threshold on raw model predictions as a final management tool to delineate suitable and unsuitable habitat. Selection of this threshold has a significant impact on suitability determinations (Nenzén & Araújo, 2011), and the appropriate threshold depends on a model's purpose and the costs of misclassification (Fielding & Bell, 1997; Pearson et al., 2004). For identifying the habitat of rare species, the cost of false negatives (i.e., misidentifying true suitable habitat as unsuitable) is much greater than the cost of false positives. Therefore, we selected a fixed sensitivity of 0.95 in thresholding our model results to ensure that areas identified as suitable encompassed a high proportion of true ERPE presences.

### 2.3 Field validation

We conducted field surveys in the fall of 2022 to assess the accuracy of the original ensemble model. These surveys were conducted by generating a series of 50‐m cells overlapping the ensemble model's suitability predictions. We systematically surveyed these cells in groups of at least three individuals by slowly walking in a straight line through cells (Williams et al., 2009; Willoughby, 2000). Where ERPE individuals were observed, we then mapped new subpopulations.

We kept records of all cells surveyed during field validation. These records were used to assess how much area was surveyed, the suitability value predicted by the ensemble model in all surveyed cells, and the cells in which new ERPE individuals were observed. Areas where no ERPE individuals were detected within these cells were considered true absence observations.

We used a Wilcoxon rank sum test to analyze whether ensemble model suitability predictions at locations where ERPE was observed differed from model predictions where ERPE was not observed. We also calculated Cliff's delta effect size because effect sizes are more meaningful for comparison than metrics such as p‐value and test statistics (Sullivan & Feinn, 2012). The Wilcoxon rank sum test and Cliff's delta were used, rather than a t‐test, because model suitability predictions were not normally distributed.

### 2.4 Final model

#### 2.4.1 Input points

We included all new species observations from field validation as input points in the final model. We also altered our selection of presence points to better include accurately mapped polygon observations of ERPE individuals. In the original model, we primarily utilized point observations of the species, with some manually generated points in mapped polygons distinct from any point observations. In the final model, we included all point observations and all accurately mapped polygons.

However, mapped polygons were very large in many cases, and considering all the area within each mapped polygon yielded an extremely high number of species presences which overwhelmed the point observations. We utilized the mapped polygons by first isolating those with an area less than 20‐m^2^. For these polygons, we generated one point in the polygon centroid. For polygons larger than 20‐m^2^, we rasterized polygons on a 10‐m resolution grid and generated one point in the centroid of each polygon cell. We then further restricted these points by filtering them so that no points were closer together than 40 meters. This two‐step approach helped to ensure smaller polygons had at least one point generated within them, but large polygons did not have hundreds of points.

The true point observations and the points generated from the mapped polygons were then merged. This set included 892 points from point observations and 655 points from mapped polygons, for a total of 1547 species observations.

True absence data were also available from the field validation of the original model because we considered surveyed areas with no ERPE observations as true absences. However, we did not want to overwhelm the model with these true absence points, which were overall very near and similar to presence points. We generated true absence points through a similar process as generating presence from mapped polygons. Starting with the set of cells surveyed in the field validation, we first masked out areas with ERPE observations, then rasterized the remaining areas at 20‐meter resolution and generated one point in each cell centroid. This yielded 2061 true absence points.

We preserved the same ratio of presence to background/absence points in the final model so that metrics such as AUC‐PR would be comparable between the original and final modeling efforts. Therefore, we had to generate additional background points to balance the increase in presence points available in the final model. These background points were randomly generated as in the original model.

Lastly, we took two new steps in filtering the final set of points before modeling. We filtered presence observations so that no observations were closer than 2 meters. This prevented some areas with many closely packed point observations from overwhelming the model. We also filtered the background and true absence points so that none were closer than 100 meters to any presence points. This step helped ensure that absence points were sufficiently dissimilar from the presence points.

With the final set of filtered presence and background/absence points we again randomly split the data into training and validation sets, with two‐thirds in the training set and one‐third in the validation set.

#### 2.4.2 Environmental covariates

All environmental covariates from the original model were included in the final model, and one new covariate was added, soil color index. During field validation, we noted that some locations identified as the Smoky Hill member of the Mancos Shale in our geology layer did not have the distinctive gray color expected in this geologic stratum. Soil textures also differed, with these soils being loamier than where ERPE is typically found.

We used satellite imagery as an additional way to identify soil color and thus the relevant geologic stratum. We downloaded a clear Landsat scene of the study area and used the red and green bands to calculate the soil color index (Mandal, 2016), calculated as: (Red − Green)/(Red + Green). The soil color index was calculated with a Landsat scene from Landsat 9 Collection 2 (USGS, 2022). Soil color index was resampled by bilinear interpolation from the original 30‐m resolution to 1‐m resolution to match the other predictors.

#### 2.4.3 Modeling

We altered our modeling approach to obtain a more diverse set of model types. In the original model effort, we employed two Random Forest (RF) implementations, BRT, and MaxEnt. RF and BRT are both tree‐based machine‐learning algorithms so their results are likely more similar than other methods. MaxEnt shares qualities of both regression‐based algorithms and machine learning algorithms (Phillips et al., 2006), but was not included in the original weighted ensemble.

Confidence in modeling results is increased when diverse models with different assumptions reach similar conclusions (Zimmer et al., 2021), so we used a more varied set of model approaches in the final model. We again ran the downsampled RF, BRT, and MaxEnt models. We also included Multivariate Adaptive Regression Splines (MARS) fit in the ‘earth’ package (Milborrow, 2019) and Generalized Additive Models (GAM) fit in ‘mgcv’ (Wood, 2015), two additional regression‐based modeling methods (Valavi et al., 2021). Unlike in the original model, we did not run the shallow random forest model from the ‘ranger’ package. The model parameters utilized are shown in Appendix S1.

We again used AUC‐PR to calculate a weighted ensemble model. This new weighted ensemble included RF, BRT, MaxEnt, MARS, and GAM. Therefore, the final ensemble was based on two tree‐based machine learning algorithms, two regression‐based methods, and one method with characteristics of each. This is much more varied than the original ensemble model, which included only three tree‐based regression models.

## 3 RESULTS

### 3.1 Original model

Validation metrics for each algorithm used in the original model were computed using the validation data held‐out from model construction. AUC‐ROC for these validation data was very similar for all models (between 0.980 and 0.989) (Table 1). AUC‐PR was more variable (from 0.618 to 0.793), with MaxEnt having the worst performance. The weighted ensemble model, which was weighted by AUC‐PR and did not include the MaxEnt model, had the highest AUC‐PR, slightly above RF Downsample. BRT had the highest score for Correlation.

To account for differences in performance across these validation metrics, we calculated a normalized average of each model's validation scores. RF Downsample had the highest score by this normalized average, followed by the weighted ensemble, BRT, RF Shallow, and MaxEnt.

### 3.2 Field validation

Field validation of the original ensemble model was carried out over near‐field days totaling approximately 112 person‐hours of sampling effort. This sampling yielded 55 new subpopulations of ERPE covering 0.01 km^2^ (2.47 acres). In total, 1.47 km^2^ (362.35 acres) was surveyed during this sampling effort.

**FIGURE 2:**
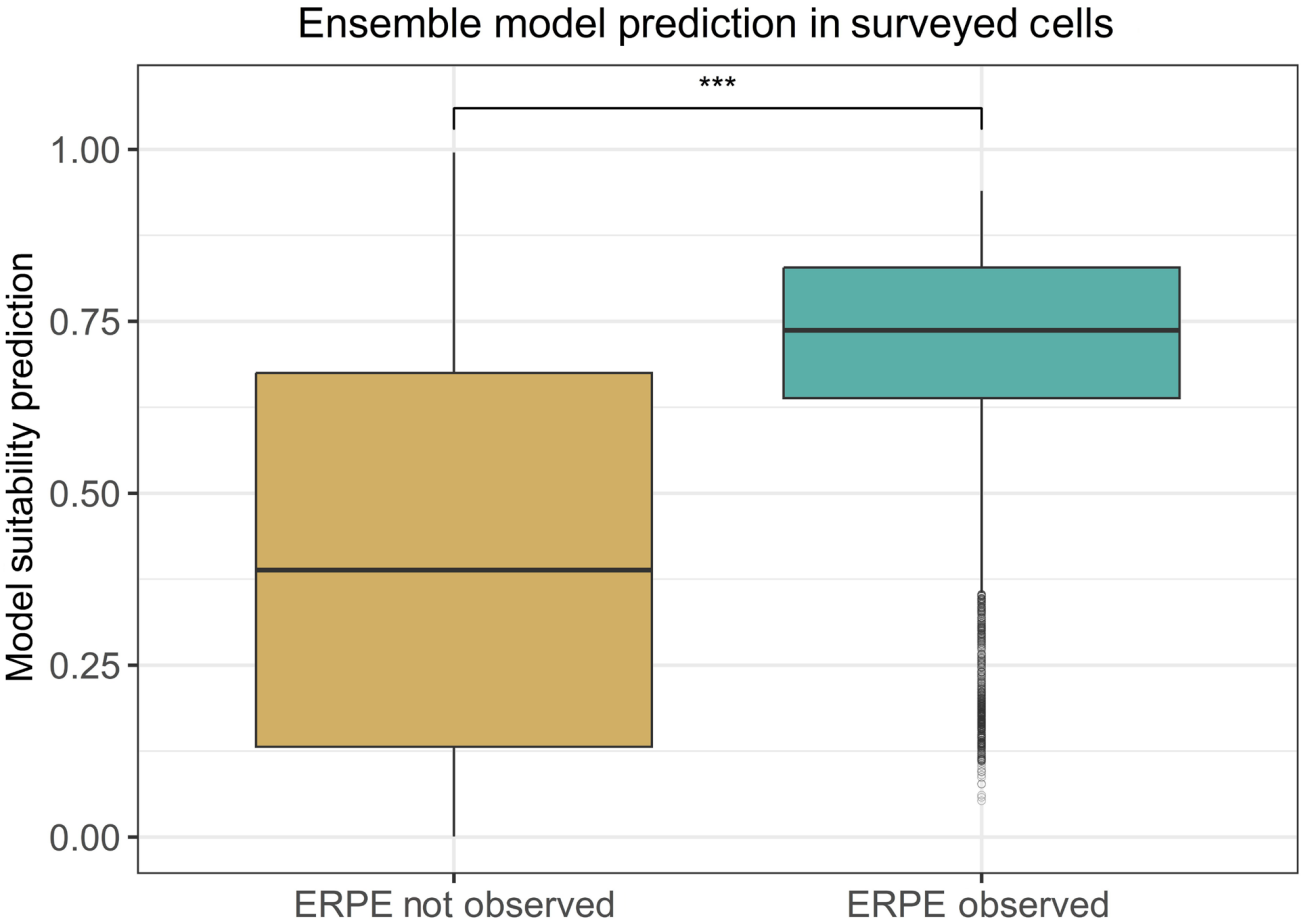
Boxplots of ensemble model predictions in cells surveyed in field validation, comparing predictions where ERPE was observed and not observed. Asterisks represent statistical significance of the Wilcoxon rank sum test (*p* < .0001 = ***).

Model suitability predictions in cells where ERPE was present and absent were significantly different from each other (Figure 2). The mean model prediction in cells where ERPE was absent was 0.411 (SD = 0.290), while the mean model prediction where ERPE was present was 0.703 (SD = 0.168). A one‐sided Wilcoxon rank sum test showed this difference was highly statistically significant (*p* < .0001), and Cliff's delta showed a large effect size (*d* = 0.575).

We ran the same statistics for each of the individual models as well as the ensemble. For Cliff's delta effect size, RF Shallow had the highest score (0.594), followed by the weighted ensemble (0.575), BRT (0.557), RF Downsample (0.549), and finally Maxent with a medium effect size (0.349). Though these effect sizes varied between models, the Wilcoxon rank sum test showed they were all highly statistically significant (*p* < .0001).

We also divided model suitability predictions into cells predicted to be suitable and unsuitable based on each model's fixed sensitivity threshold of 0.95, and performed a Chi‐square test comparing the number of cells in each of these classes where ERPE was observed and not observed. In the original ensemble model, the prediction value corresponding to this sensitivity was 0.388. This analysis also showed a statistically significant difference, with ERPE observed in 1.27% of the cells predicted to be suitable, and 0.09% of cells predicted to be unsuitable (Appendix S1: Tables S1 and S2).

### 3.3 Final model

Compared to the original ensemble model, performance of the final ensemble model was improved in all the validation metrics (Table 2). AUC‐PR rose from 0.793 to 0.825, Correlation rose from 0.599 to 0.663, and AUC‐ROC rose very slightly from 0.989 to 0.990.

**TABLE 2:** Validation metrics of models in the final model effort.

	AUC‐ROC	AUC‐PR	Correlation	Normalized average
RF	0.991	**0.845**	0.619	0.917
BRT	**0.992**	0.844	**0.682**	**0.999**
MaxEnt	0.980	0.727	0.679	0.792
MARS	0.967	0.684	0.654	0.597
GAM	0.960	0.347	0.392	0
Weighted Ensemble	0.990	0.825	0.663	0.942

*Note*: The bolded number in each column denotes the model with the highest score for that validation metric. Normalized average was calculated by scaling the data range of each validation metric from zero to one, then averaging the scaled result of the three metrics for each model.

**FIGURE 3:**
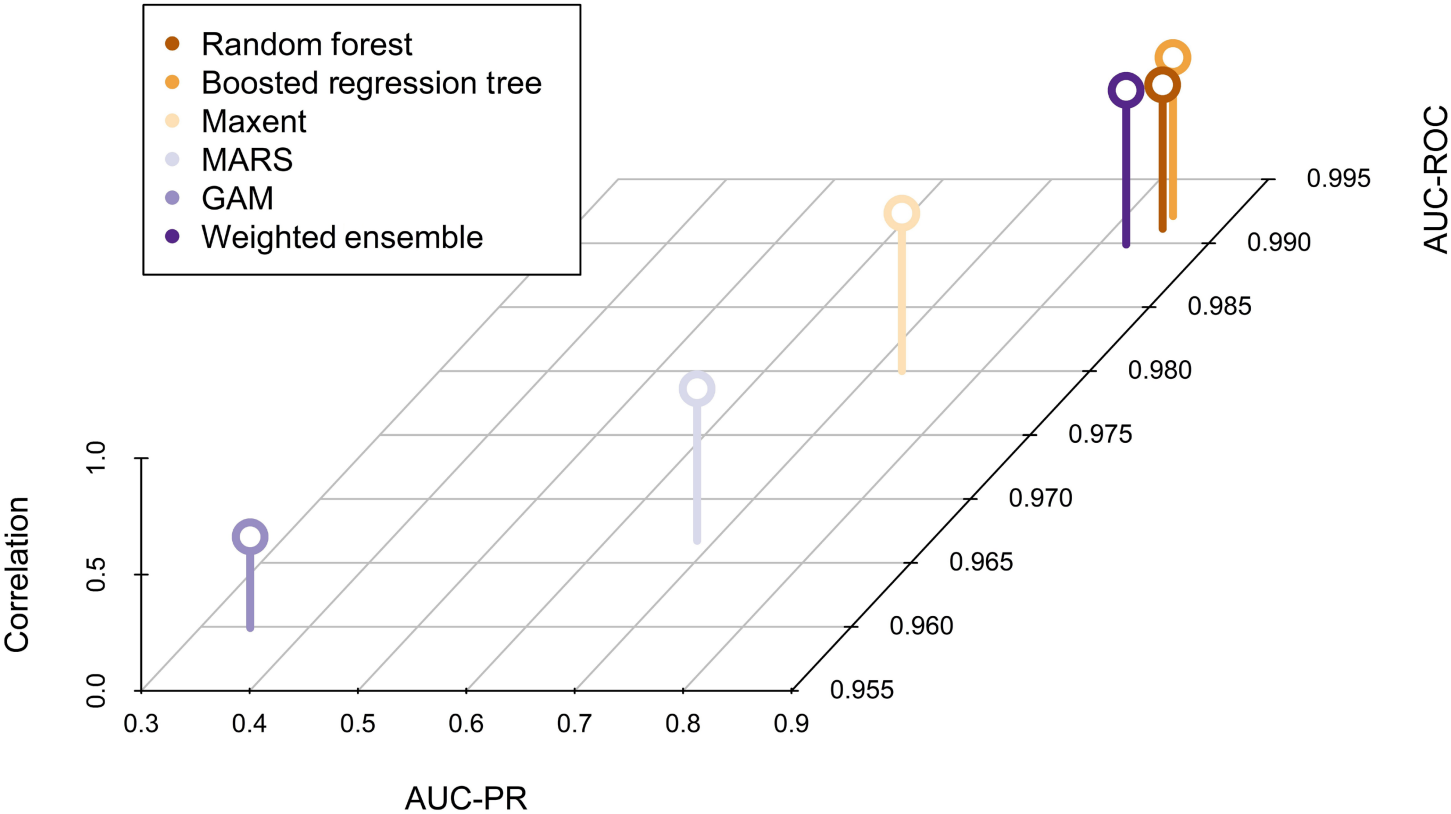
Three‐dimensional scatterplot of final model validation metrics.

The weighted ensemble model was similar to the individual models in all metrics (Table 2; Figure 3). Though the ensemble was not best in any metric, it had the second‐highest normalized average of the three metrics. The GAM model had the lowest performance for all metrics.

The models calculated relative variable importance differently. To compare variable importance between them, we determined the ranked variable importance from each model (Table 3). Some relationships are evident, with elevation and geology among the most important variables for all models. Precipitation was the most important variable in three of the five models, and eastness was the least important variable in all models.

**TABLE 3:** Relative variable importance rank from each model.

	Precipitation	Elevation	Slope	Northness	Eastness	Geology class	Soil color index
RF	1	2	5	6	7	3	4
BRT	1	3	5	6	7	2	4
Maxent	3	2	5	6	7	1	4
MARS	1	*NA*	*NA*	*NA*	*NA*	2	3
GAM	6	3	4	4	7	1	2

*Note*: MARS and GAM models have variable interaction terms, but these are not shown. For RF, the Mean Decrease GINI rank is shown. *NA* indicates variables not utilized in the MARS model in non‐interaction terms.

The MARS model evaluated all variable interactions and discarded many individual variables and interaction terms. Only precipitation, geology, and soil color index were utilized in the MARS model in non‐interaction terms. However, all variables besides eastness were utilized in interaction terms. For example, the second‐most important variable was the interaction of elevation, slope, soil color index, and precipitation, but elevation and slope were not utilized in the model as individual terms. This model feature “pruning” is a feature of MARS models (Kartal & Bozdogan, 2015; Milborrow, 2019) and simple variable importance tests showed all variables were important, though variable selection prior to modeling could potentially have improved fit.

### 3.4 Habitat characteristics

**FIGURE 4:**
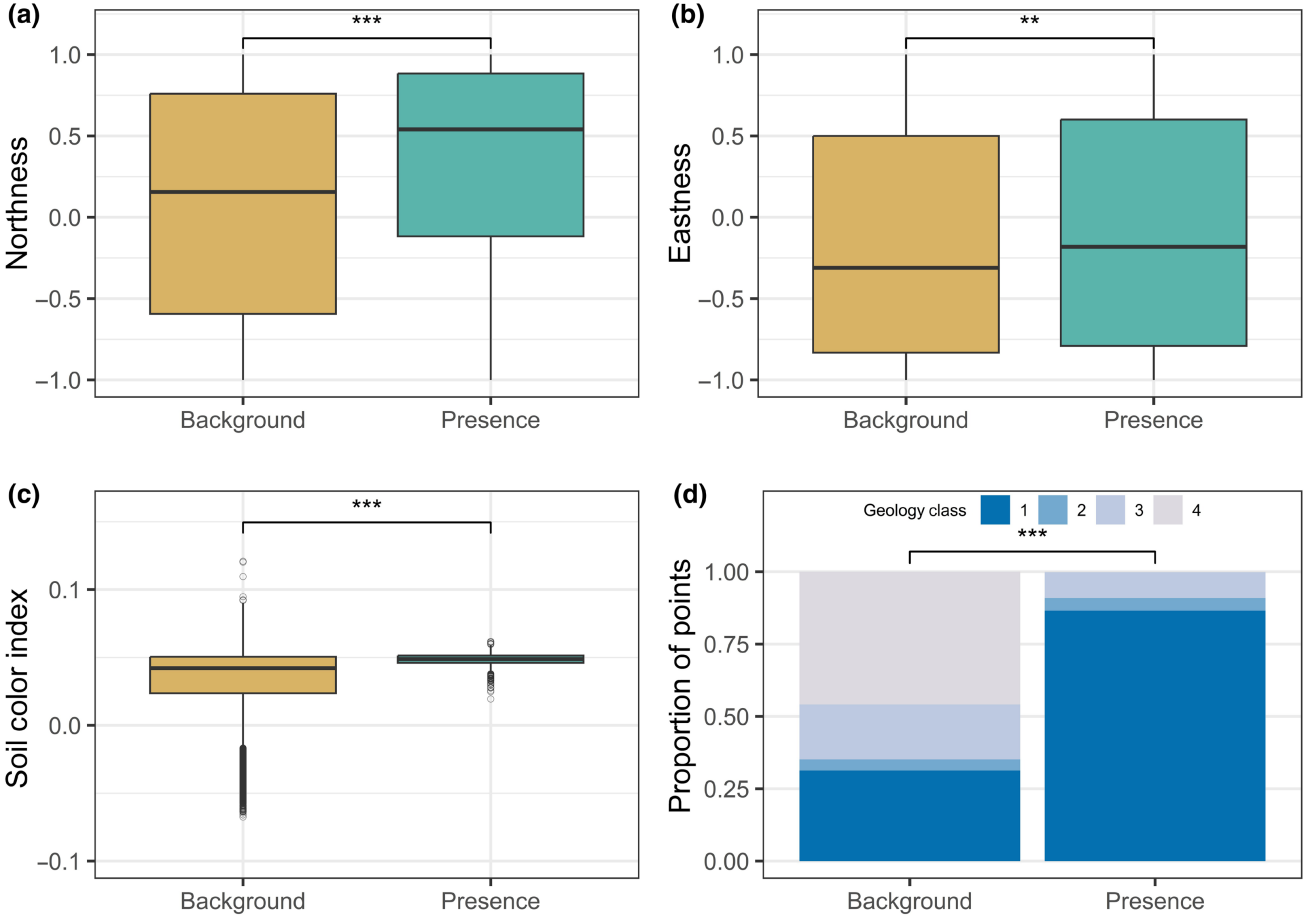
Comparisons of environmental covariates at the presence points and background points used in the final model. Asterisks represent statistical significance of the Wilcoxon rank sum test (*p* < .01 = **; *p* < .0001 = ***).

Comparing environmental covariates at the presence points and background points used in the final model reveals several important habitat characteristics of ERPE (Figure 4). Geology had the strongest association with ERPE presence. Mean geology class at presence points was 1.23, compared to 2.81 at background points. This difference was strong and statistically significant (Cliff's *d* = −0.618, *p* < .0001). For ERPE presence points, 86.55% were in geology class 1, meaning they were within the Smoky Hill member or within 1 meter of it, while only 30.77% of background points were in class 1.

ERPE presence was associated with significantly more north‐facing aspects, with mean northness of 0.33 at presence points, compared to 0.08 at background points (Cliff's *d* = 0.204, *p* < .0001). ERPE presence points were on slightly more east‐facing slopes, with a mean of −0.10 compared to −0.16 at background points. This was a weak but statistically significant association (Cliff's *d* = 0.048, *p* = .0014). Mean soil color index at ERPE presence points was 0.05 compared to 0.03 at background points. This was a strong association despite the small magnitude of difference (Cliff's *d* = 0.357, *p* < .0001).

### 3.5 Habitat suitability predictions

For estimating suitable and unsuitable ERPE habitat from raw model predictions, we applied a fixed sensitivity threshold of 0.95 to model predictions. In the original model, the prediction value corresponding to this sensitivity was 0.388. Therefore, 95% of species presences should be correctly identified at a cutoff value of 0.388 delineating suitable and unsuitable. Our field validation of the model showed that this was extremely accurate—of the locations where we observed ERPE (i.e., true presences), 93.69% had a model prediction greater than the cutoff value. Specificity at this 95% sensitivity threshold was 0.941, meaning 94.1% of absences should be correctly identified with this cutoff value. However, in our field validation, we found only 49.94% of absences were correctly identified.

In the final model, the prediction corresponding to the 0.95 sensitivity threshold was 0.330. Setting the prediction cutoff of the models to this 0.95 sensitivity threshold ensured that sensitivity of the models was high, but specificity remained high as well (0.941 for the original model, and 0.934 for the final model). A high sensitivity was necessary for our purpose of identifying potential habitat of a rare species. Choosing the prediction threshold where sensitivity plus specificity is maximized is often suggested (Liu et al., 2013), but we preferred the more easily interpretable and consistent threshold of a fixed 95% sensitivity.

**FIGURE 5:**
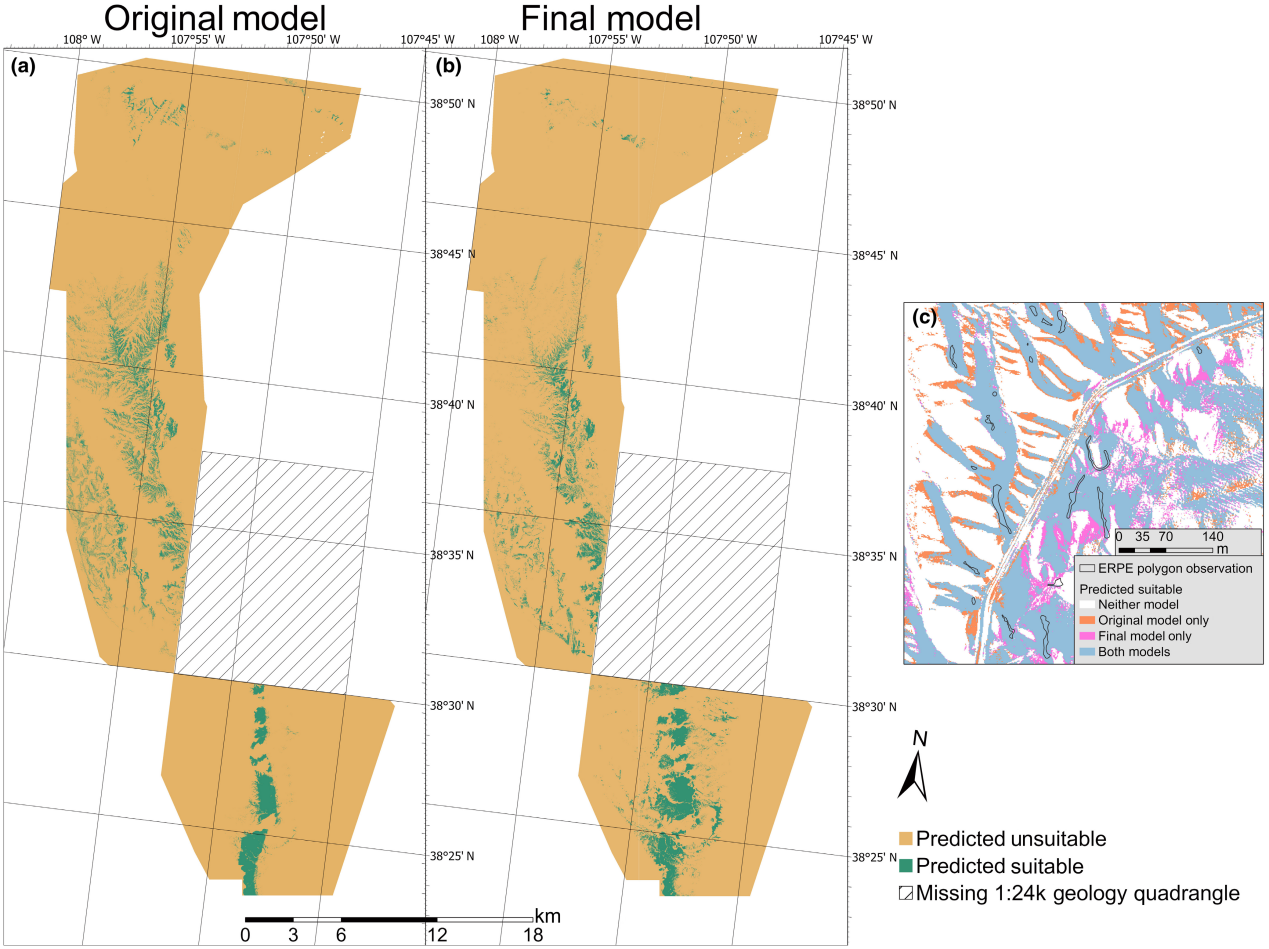
Maps comparing predicted habitat suitability from the original (a) and final (b) ensemble models. Raw model predictions have a 95% sensitivity threshold applied to delineate suitable and unsuitable habitat. The inset map (c) shows close‐up model results in one location, comparing areas with predicted habitat suitability from each model.

When applied to the raw model predictions, the fixed 95% sensitivity threshold yielded similar determinations of suitable habitat and a similar percentage of the study area being identified as suitable (Figure 5). The cutoff showed 6.04% of the study area was suitable in the original model, and 6.48% was suitable in the final model. For other commonly used prediction thresholds, the cutoff prediction values and suitable habitat percents were also similar between the models (Appendix S1: Table S3).

For the final model, this translated to a total of 33.66 km^2^ of suitable ERPE habitat (8318 acres). Of this area, however, only 12.98 km^2^ (3208 acres) occurs on BLM land. The remaining habitat is on private lands, much of which are currently agricultural fields or adjacent to fields, and not thought to support ERPE populations.

## 4 DISCUSSION

### 4.1 Species habitat

We used a 1‐meter resolution LiDAR‐derived DEM in our model, and derived northness, eastness, and slope from this DEM. Few analyses have used LiDAR in species distribution models, though see Ackers et al. (2015), Keppel et al. (2017), and Questad et al. (2014). For species with highly specific habitats, higher resolution data from LiDAR and other remote sensing methods (He et al., 2015) should provide the best opportunity to accurately capture fine‐scale microtopography such as the small swales where *E. pelinophilum* is often found. Our high‐resolution predictions also capture habitat suitability at a scale relevant to the species' patch size (Gogol‐Prokurat, 2011).

Our analysis confirmed that *E. pelinophilum* is very strongly associated with a single geologic stratum, the Smoky Hill member of the Mancos Shale, and availability of a high‐resolution 1:24 k geology mapping allowed accurate identification of this stratum. However, one geology quadrangle with known ERPE populations was not available at this resolution and was not included in our modeling (Figure 5). Additional mapping to complete this survey would allow us to make predictions in this missing portion of the species' habitat.

### 4.2 Model validation

Appropriate model validation remains an issue in species distribution models, with many metrics used and the most appropriate metric depending on characteristics like species rarity (Sofaer et al., 2019). We evaluated only threshold‐independent metrics including AUC‐ROC, AUC‐PR, and Correlation. We considered AUC‐PR to be the most important metric, and removed the MaxEnt model from inclusion in the original weighted ensemble due to its somewhat lower AUC‐PR. However, closer scrutiny after the final modeling showed the original MaxEnt model had the second‐highest correlation, suggesting MaxEnt may have been a positive contribution to the original weighted ensemble.

In the final model effort, no model performed best in all validation metrics, demonstrating the tradeoffs and differences in performance between these metrics. The weighted ensemble had the second‐highest normalized average of all metrics, but was not best in any one metric. Ambiguity in individual model performance and validation metrics is a primary argument for considering ensemble models (Araújo & New, 2007), though the performance improvement of ensemble species distribution models in general remains unclear (Hao et al., 2019). However, ensemble models have been promoted for at‐risk species in particular due to their ability to reduce uncertainty between models (Ramirez‐Reyes, Street, et al., 2021).

### 4.3 Field validation

Field validation of the original model provided a secondary form of model validation. Direct validation of species distribution models remains extremely rare (Araújo & Guisan, 2006; Fois et al., 2018), but we believe this provides crucial information about a model's predictive capabilities (Tredennick et al., 2021) and increases confidence in results. We found model suitability predictions were very strongly associated with ERPE observation for all models (Figure 2; Appendix S1: Table S2). The weighted ensemble model was the model with the highest AUC‐PR, and the second‐highest Cliff's d effect size and Cramer's V effect size comparing prediction values where ERPE was observed and not. AUC‐PR appeared to be the validation metric most predictive of a model's ability to make suitability predictions associated with new observations in the field, but we found it was not perfectly associated with the field validation performance in our limited sample.

Field validation helped us find a new variable to include in the final model, the soil color index. This represented a biologically relevant improvement since some soils we observed during field validation were beige‐colored, with a loamier texture and substantially lower clay content than soils ERPE is known to occupy. This microsite variation was not captured in the geology mapping. However, the soil color index variable helped to distinguish this variation and was an important contribution to the final models, with greater variable importance than northness, eastness, and slope.

In field validation, we found that 93.69% of new ERPE observations were correctly identified using the 95% sensitivity value to delineate suitable and unsuitable species habitat. This is extremely close to the expectation that 95% of true presences should be correctly identified at this level. Model specificity at this prediction value was 0.941, meaning 94.1% of true species absences should be correctly identified, but field validation showed only 49.94% of absences were correctly identified. This difference is likely due to our field validation taking place very near to species presences in generally high suitability areas, rather than at the edges of the study area. This likely led to spatial autocorrelation between the species presence data used to train the model and the cells surveyed in the field validation. Areas surveyed in validation were between 25 and 2000 m from known species presences, but much of the surveyed area was closer than 200 m from known presences. This increased the likelihood of surveyed cells having habitat conditions similar to those of species presences, favoring model misclassification of species absences. This was somewhat unavoidable given the limited spatial scale of the study area, but surveying some locations farther from known presences may have limited this effect.

### 4.4 Management implications

The original and final models help more definitively distinguish the potential occupied habitat of clay‐loving wild buckwheat (*E. pelinophilum*). This information has numerous benefits for the Bureau of Land Management, and directly addresses BLM conservation priorities for special status species. The BLM's Strategic Plan for Special Status Species Conservation and Recovery specifically states “science‐related activities (e.g., research, inventory, monitoring, and habitat models) should be directly related toward the implementation of on‐the‐ground conservation and recovery efforts” (BLM, 2022b) to prioritize the Endangered Species Act section 7(a)(1) proactive recovery mandate.

Our models identify where *E. pelinophilum* may be threatened by existing land use activities and new roads, off‐highway recreation, or other developments, and can help redirect these developments when feasible or inform where additional surveys are needed before projects can be initiated. The models help inform survey efforts to focus on areas where the species is most likely to occur, maximizing survey effort and reducing costs while also increasing the likelihood of detecting previously undocumented populations. Our results definitively quantify and greatly refine species managers' understanding of how narrowly restricted suitable habitat for the species is within its range. This has greatly informed the USFWS Species Status Assessment process and informed their decision‐making regarding the status of *E. pelinophilum* under the Endangered Species Act.

Lastly, separate analyses have documented that *E. pelinophilum* has experienced significant population declines range‐wide in recent years. Long‐term monitoring sites indicate a 70% reduction in mature individuals throughout the range between 2017 and 2022, likely due to persistent drought (BLM, 2022a). The severity of current drought conditions has been most impacting in lower elevations, overlapping *E. pelinophilum* habitat. With the possibility of extirpation in some portions of the range, our models can also be used to identify potential areas for *E. pelinophilum* restoration, since areas with high habitat suitability offer the best possibility for successful restoration.

## 5 CONCLUSION

We constructed a series of species distribution models and a weighted ensemble model of an endangered species in western Colorado, clay‐loving wild buckwheat. The inclusion of a LiDAR‐derived DEM and a high‐resolution mapping of geologic strata helped to fit models with high accuracy. We validated our weighted ensemble model in the field, finding 55 new subpopulations of the species and demonstrating that new species observations were strongly associated with model suitability predictions and that the model can be used to guide species surveys. With the new species observations and additional information learned during the original modeling effort and field sampling, we then further refined the models and marginally improved model performance. The model outputs have direct management implications for the Bureau of Land Management, as they can be used to identify conflicts with land use activities and potential restoration sties and inform additional survey efforts.

## AUTHOR CONTRIBUTIONS

**Scott N. Zimmer:** Conceptualization (lead); data curation (lead); formal analysis (lead); methodology (lead); resources (lead); software (lead); supervision (equal); validation (lead); visualization (lead); writing – original draft (lead); writing – review and editing (lead). **Kenneth W. Holsinger:** Conceptualization (equal); data curation (equal); formal analysis (supporting); methodology (supporting); supervision (equal); validation (equal); visualization (supporting); writing – original draft (supporting); writing – review and editing (supporting). **Carol A. Dawson:** Methodology (supporting); supervision (equal); validation (equal); writing – review and editing (supporting).

## CONFLICT OF INTEREST STATEMENT

The authors declare no competing interests.

### OPEN RESEARCH BADGES

This article has earned an Open Materials badge for making publicly available the components of the research methodology needed to reproduce the reported procedure and analysis. All materials are available at https://doi.org/10.5061/dryad.dfn2z357c.

## Supporting information


Appendix S1

Appendix S2

## Data Availability

The data that support the findings of this study are openly available for download from Dryad at: https://doi.org/10.5061/dryad.dfn2z357c.
