# Supplementary material for: A field‐validated ensemble species distribution model of Eriogonum pelinophilum, an endangered subshrub in Colorado, USA
Source: Ecol Evol. 2023 Dec 14;13(12):e10816. doi: 10.1002/ece3.10816 (PMC10721943; doi:10.1002/ece3.10816)
Supplement: Supplementary file 1 — Appendix S1 [file ECE3-13-e10816-s002.docx]

# Appendix S1

## Model Parameters

Random Forest was fit with 1000 trees. Number of variables randomly selected at each split (“mtry”) was 3. Sample sizes were limited to 991 presence and 991 absence points per tree.

Boosted Regression Tree was tuned using the procedure described in Valavi et al. (2022). The following settings were used: The model was fit with 1650 trees, family was Bernoulli, tree complexity was 10, learning rate was 0.01, bag fraction was 0.5, and site weights were 1 for the presence points and the prevalence rate for background points (0.0229).

Maxent was also tuned using the procedure from Valavi et al. (2022). The arguments used in the model were: Beta multiplier = 0.25, No Auto Feature, and No Threshold (LQHP).

MARS was fit using all input variables and their interaction terms.

GAM was fit using all input variables and their interaction terms. The model was fit with a binomial distribution with a logit link. Site weights were 1 for the presence points and the prevalence rate for background points (0.0229). The smoothing parameter estimation method was REML.

## Chi-square Test of Field Validation

For the Chi-square we split the model predictions into cells which were predicted to be suitable and unsuitable for the ensemble model, based on the model’s 95% sensitivity threshold value. This value was 0.388 for the ensemble. We then calculated the number of cells in in the predicted suitable and unsuitable classes where ERPE was observed and not observed in field survey (Table S1).

The proportion of these classes where ERPE was observed was significantly different than the classes where ERPE was not observed (*Cramér’s V* *= 0.072*, *p < 0.0001*, *χ^2^= 7572.9*, *df = 1*), indicating that ERPE observation was strongly associated with the model predictions. ERPE was observed in 1.27% of the surveyed cells which were predicted to be suitable, but only in 0.09% of surveyed cells not predicted to be suitable.

**Table S1: Number of cells surveyed in field validation that were predicted to be suitable and unsuitable based on the 95% sensitivity threshold, split by where new ERPE individuals were observed and not observed.**

|  | **Cells With**  **ERPE Observed** | **Cells Without ERPE Observed** |
| --- | --- | --- |
| **Predicted Suitable (0.388 - 1)** | 9,374 | 729,030 |
| **Predicted Unsuitable (0 – 0.388)** | 631 | 727,343 |

We ran the Chi-square test for each model, separating model predictions into suitable and unsuitable prediction based on each model’s respective 95% sensitivity threshold. We then ran the Chi-square statistics and effect sizes for each of the individual models (Table S2). For Cramér’s V, RF Downsample had the highest score, followed by the weighted ensemble and BRT. Maxent had the lowest effect size, but was close to RF Shallow.

**Table S2: Comparison of Cramér’s V effect sizes for all models, comparing model predictions where ERPE was observed and not. Bolded number represents the highest score.**

|  | **Cramér’s V Effect Size** |
| --- | --- |
| **RF Downsample** | **0.075** |
| **RF Shallow** | 0.066 |
| **BRT** | 0.072 |
| **MaxEnt** | 0.060 |
| **Weighted Ensemble** | 0.072 |

## Varying Model Thresholds

**Table S3: Model suitability prediction cutoff values at four thresholds. In parentheses is the total percent of the study area identified as suitable habitat using that cutoff value.**

|  | **Fixed 95% Sensitivity** | **Maximum Specificity + Sensitivity** | **Equal Specificity and Sensitivity** | **Maximum Kappa** |
| --- | --- | --- | --- | --- |
| **Original Ensemble** | 0.388 (6.04%) | 0.542 (3.74%) | 0.411 (5.58%) | 0.872 (0.67%) |
| **Final Ensemble** | 0.330 (6.48%) | 0.436 (3.67%) | 0.356 (5.58%) | 0.815 (0.63%) |
