# Supplementary material for: A field‐validated ensemble species distribution model of Eriogonum pelinophilum, an endangered subshrub in Colorado, USA
Source: Ecol Evol. 2023 Dec 14;13(12):e10816. doi: 10.1002/ece3.10816 (PMC10721943; doi:10.1002/ece3.10816)
Supplement: Supplementary file 2 — Appendix S2 [file ECE3-13-e10816-s001.docx]

A field-validated ensemble species distribution model of Eriogonum pelinophilum, an endangered subshrub in Colorado, USA

– ODMAP Protocol –

2023-06-28

## Overview

#### Authorship

Contact :

#### Model objective

Model objective: Mapping and interpolation

Target output: Suitable vs. unsuitable habitat

#### Focal Taxon

Focal Taxon: Clay-loving wild buckwheat (Eriogonum pelinophilum)

#### Location

Location: Western Colorado, United States

#### Scale of Analysis

Spatial extent: -108.00, -107.70, 38.38, 38.84 (xmin, xmax, ymin, ymax)

Spatial resolution: 1-m

Temporal extent: Fall 2022

Temporal resolution: Single time

Boundary: natural

#### Biodiversity data

Observation type: standardised monitoring data, field survey

Response data type: presence-only, presence/absence

#### Predictors

Predictor types: climatic, edaphic, topographic

#### Hypotheses

Hypotheses: Hypotheses: Climate, topography, and geologic stratum are correlated with species presence

#### Assumptions

Model assumptions: We assumed that the species is at pseudo-equilibrium with its environment, surveys had adequately covered the species' range, species observations collected over an extended period (30-40 years) were reliable currently, and relevant ecological drivers (or proxies) of species distributions are included.

#### Algorithms

Modelling techniques: randomForest, brt, maxent, mars, gam

Model complexity: Non-linear and interacting predictors to allow for more realistic cause-effect representation. Random Forest and Boosted Regression Tree were limited to avoid overfitting. MARS and GAM included all variable interactions.

Model averaging: Yes. An ensemble model weighted by each model's AUC-PR was evaluated.

#### Workflow

Model workflow: Model calibration done with careful consideration of data limitations. Models were evaluated with AUC-ROC, AUC-PR, and correlation.

#### Software

Software: R 4.1.2. Packages used in modeling included: terra, raster, randomForest, ranger, dismo, earth, mgcv.

Code availability: N/A

Data availability: Will be made available in an open, online, digital repository.

## Data

#### Biodiversity data

Taxon names: Eriogonum pelinophilum

Taxonomic reference system: Flora of North America Editorial Committee. 2005. Flora of North America North of Mexico. Vol. 5. Magnoliophyta: Caryophyllidae: Caryophyllales, Polygonales, and Plumbaginales. Oxford Univ. Press, New York. vii + 656 pp.

Ecological level: species

Data sources: Bureau of Land Management surveys

Sampling design: Subjective spatial sampling (representative sampling of plant communities; once-off temporal sampling

Sample size: 1,547 points of species presence. 892 points were collected as points, 655 points were generated from within collected polygon observations

Scaling: LiDAR data were available at 1-m resolution, other variables were resampled to 1-m

Absence data: True absence data was available following field surveys of the original model

Background data: Randomly generated background points throughout the study area

Errors and biases: Subjective sampling locations, background points not generated with a bias grid

#### Data partitioning

Training data: Two-thirds (66.66%)

Validation data: One-third (33.33%)

Test data: Truly independent data were obtained by conducting a systematic survey for new species occurrences and comparing occurrences with model predictions

#### Predictor variables

Predictor variables: Elevation, slope, northness, eastness, 30-year normal precipitation, geologic stratum, soil color index

Data sources: LiDAR, LiDAR, LiDAR, LiDAR, PRISM, Colorado Geological Survey mapping, Landsat

Spatial extent: -108.00, -107.70, 38.38, 38.84 (xmin, xmax, ymin, ymax)

Spatial resolution: 1m, 1m, 1m, 1m, 800m, polygon, 30m

Coordinate reference system: Albers equal area North American Datum 1983; +proj=aea +lat_0=23 +lon_0=-96 +lat_1=29.5 +lat_2=45.5 +x_0=0 +y_0=0 +datum=NAD83 +units=m +no_defs +type=crs

Temporal extent: 30-year normal precipitation from 1991-2020

Data processing: Slope and aspect were calculated from elevation using the "terrain" function in the "raster" package in R, using 8 cell neighbors (queen case). Aspect, in degrees, was used to calculate northness and eastness: Northness = cos (aspect * π / 180); Eastness = sin (aspect * π / 180). Soil color index was calculated using the Landsat bands Red and Green: Soil color index = (Red - Green) / (Red + Green), see (Mandal, U. 2016. Spectral color indices based geospatial modeling of soil organic matter in Chitwan District, Nepal. ISPRS - International Archives of the Photogrammetry, Remote Sensing and Spatial Information Sciences XLI-B2:43–48.). Raw data were resampled by nearest neighbor interpolation to 1-m resolution.

## Model

#### Variable pre-selection

Variable pre-selection: Ecological pre-selection of variables we deemed important for the species, and variable importance was checked with “Boruta” package variable selection.

#### Multicollinearity

Multicollinearity: Not accounted for. Elevation and precipitation were highly correlated (r = 0.95), but the next-highest correlation was very low (0.29). Our models were predominantly tree-based, and the non-tree based models included all variable interaction terms.

#### Model settings

randomForest: ntree (1000), mtry (3)

brt: distribution (Bernoulli), nTrees (1650), interactionDepth (10), shrinkage (0.01), bagFraction (0.5)

maxent: featureSet (LQHP), regularizationMultiplierSet (0.25)

mars: formula (species presence ~ eastness * northness * elevation * geologic stratum * soil color index * slope * precipitation)

gam: family (Binomial), formula (species presence ~ eastness * northness * elevation * geologic stratum * soil color index * slope * precipitation), smoothTerms (REML), weights (1 for species presence, the prevalence rate for background points (0.0229))

#### Model selection - model averaging - ensembles

Model ensembles: Weighted ensemble model. The AUC-PR of each model was added, then the AUC-PR of each model was divided by the AUC-PR sum to get the model's weight. Model outputs were multiplied by the model weight, then added together to get the weighted ensemble.

#### Threshold selection

Threshold selection: We selected a fixed sensitivity threshold of 0.95 to threshold results. We selected this value because maximizing true presences was most important for our model of a rare species' habitat.

## Assessment

#### Performance statistics

Performance on training data: AUC, Correlation

Performance on validation data: AUC, Correlation

Performance on test data: t-test, effect size, chi-square

#### Plausibility check

Expert judgement: Model predictions were systematically surveyed in the field. New species occurrences discovered during field sampling were significantly correlated with model predictions.

## Prediction

#### Prediction output

Prediction unit: We used continuous predictions of habitat suitability, as well as binary suitable / unsuitable determinations from thresholding predictions based on a 95% fixed sensitivity.
